# Amylomaltases in Extremophilic Microorganisms

**DOI:** 10.3390/biom11091335

**Published:** 2021-09-09

**Authors:** Claudia Leoni, Bruno A. R. Gattulli, Graziano Pesole, Luigi R. Ceci, Mariateresa Volpicella

**Affiliations:** 1 Institute of Biomembranes, Bioenergetics and Molecular Biotechnologies, Consiglio Nazionale delle Ricerche, Via Amendola, 70126 Bari, Italy; c.leoni@ibiom.cnr.it (C.L.); b.gattulli@ibiom.cnr.it (B.A.R.G.); graziano.pesole@uniba.it (G.P.); 2 Department of Biosciences, Biotechnologies and Biopharmaceutics, University of Bari Aldo Moro, Via Amendola 165/A, 70126 Bari, Italy

**Keywords:** amylomaltases, 4-α-glucanotransferases, starch modification, extremophilic microorganisms, *Bacteria*, *Archaea*

## Abstract

Amylomaltases (4-α-glucanotransferases, E.C. 2.4.1.25) are enzymes which can perform a double-step catalytic process, resulting in a transglycosylation reaction. They hydrolyse glucosidic bonds of α-1,4′-d-glucans and transfer the glucan portion with the newly available anomeric carbon to the 4′-position of an α-1,4′-d-glucan acceptor. The intramolecular reaction produces a cyclic α-1,4′-glucan. Amylomaltases can be found only in prokaryotes, where they are involved in glycogen degradation and maltose metabolism. These enzymes are being studied for possible biotechnological applications, such as the production of (i) sugar substitutes; (ii) cycloamyloses (molecules larger than cyclodextrins), which could potentially be useful as carriers and encapsulating agents for hydrophobic molecules and also as effective protein chaperons; and (iii) thermoreversible starch gels, which could be used as non-animal gelatin substitutes. Extremophilic prokaryotes have been investigated for the identification of amylomaltases to be used in the starch modifying processes, which require high temperatures or extreme conditions. The aim of this article is to present an updated overview of studies on amylomaltases from extremophilic *Bacteria* and *Archaea*, including data about their distribution, activity, potential industrial application and structure.

## 1. Introduction

Amylomaltases are prokaryotic 4-α-glucanotransferases (4α-GTases) of the CAZy glycoside hydrolase (GH) family GH77 [1], accessed on 3 Sepetember 2021. This family contains only enzymes of the E.C. 2.4.1.25 class, detected both in prokaryotes (amylomaltases) and in plants and algae (named disproportionating enzymes, or D-enzymes). It should be noted that 4α-GTases are not unique to the GH77 family, as they can also be detected, with different sequence characteristics, in the GH13 and GH57 families. The 4α-GTase enzyme catalyses the hydrolysis of glucosidic bonds in α-1,4′-d-glucans and the transfer of the glucoside with the newly formed reducing end to the 4′-position of an α-1,4′-d-glucan acceptor. Details on the distribution in nature of 4α-GTases, their requirements for donor and acceptor substrates and the resulting products have been already reviewed [2]. The transferase reaction can also be intramolecular, leading to the synthesis of circular molecules composed of six to eight glucose units, known as cyclodextrins (CDs), and cycloamyloses (CAs), which are generally large-ring cyclodextrins with a degree of polymerization (DP) from sixteen to several hundred glucose units [2,3]. Low molecular weight CAs with 5–7 DP have also been detected and will be reported in this review. D-enzymes are involved in the synthesis of CDs, while amylomaltases produce the larger CAs.

Amylomaltases in prokaryotes have been associated with different functions. In *Escherichia coli*, the enzyme is required for bacterial growth on maltooligosaccharides and its gene is inducible by maltose [4]. The products of the enzymatic reaction are glucose (required for glycolysis) and longer maltooligosaccharides from which glucose-1-phosphate is produced by the action of a maltodextrin phosphorylase. The genes for amylomaltase (*MalQ*) and glucan phosphorylase (*MalP*) constitute the malPQ operon. Recently, the involvement of amylomaltase in the degradation of glycogen was also demonstrated in *E. coli* [5]. Other functions of amylomaltases have also been indicated. In the chemolytoautotrophic bacterium *Aquifex aeolicus*, the enzyme might be involved in the metabolism of glycogen, since its gene is located in the glycogen operon and the bacterium lacks the gene for the maltose transport proteins [6]. In *Corynebacterium glutamicum*, it has been postulated that amylomaltase may be involved in the synthesis of trehalose. The sugar is required by the bacterium to counteract osmotic stress and for the production of mycolic acids, important components of the cell envelope in *Corynebacteriaceae* [7,8].

The interest in amylomaltases is due to their ability to modify starch and the consequent wide range of applications in the food and pharmaceutical fields. In this regard, several studies have been carried out on amylomaltases from extremophiles as potentially more robust enzymes, better suited for industrial processes. Here, we review and describe the results of research on amylomaltases from extremophiles. Structural data are also compared with those of amylomaltases from mesophilic bacteria to highlight differences between the enzymes, which are possibly related to their different activity in extreme conditions. This analysis may be of interest for studies aimed at understanding the structure/activity relationships for this class of enzymes and, possibly, lays the groundwork to design amino acid mutations for further enzyme optimization.

## 2. Distribution of Amylomaltases in Extremophiles

Currently (as of September 2021) the GH77 family counts 12,560 entries of bacterial amylomaltases, 76 entries of archaeal enzymes and 94 entries from plants and algae. In extremophiles, the number of characterized amylomaltases is limited (Table 1), even if thousands of potential coding genes are nowadays available in databases of genome sequences, providing a large resource of potential useful enzymes to exploit. The identification of enzymes reported in Table 1 derives from an analysis of public databases (CAZy, Uniprot, PDB and PubMed). With the exception of a single enzyme from the halophile *Haloquadratum walsbyi* (NCBI acc. Number MZ422727), all the amylomaltases reported in Table 1 are from thermophilic or hyper-thermophilic microorganisms. Entries are further subdivided according to the domain of competence (*Archaea* or *Bacteria*). The two enzymes from *Thermus thermophilus* differ by a single amino acid and refer to two different strains: HB8 for Q5SIV3 and AT-62 for O87172. It must be also noted that the enzyme with the Uniprot accession number O87172 was originally thought to belong to *T. aquaticus* [9], but currently it is assigned to *T. thermophilus*. Additionally, in the GenBank entry of the related nucleotide sequence (AB016244) the note “originally submitted as *Thermus aquaticus*” can be found. Therefore, in this review we will always indicate *T. thermophilus* as the organism from which the gene was isolated. For each entry a 3D structure was indicated. In the cases for which the 3D structure was not available, Phyre2 [10] was used for the development of a structural model (this is indicated by the PDB entry used as the template structure, followed by a subscript P). All models have 100% confidence.

## 3. Activity and Applications of Extreme Amylomaltases

**Figure 1:**
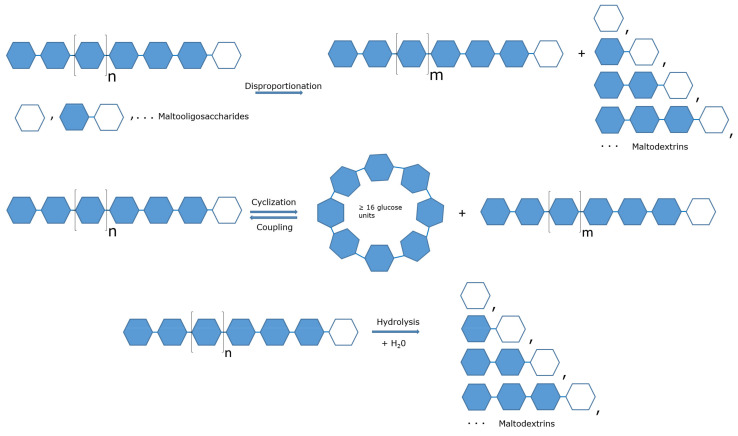
Typical reactions catalyzed by 4α-GTases. Disproportionation: the enzyme catalyzes the hydrolysis of a glucosidic bond in the donor α-1,4′-d-glucan and the transfer of the glucoside with the newly formed reducing end to the 4′ position of an acceptor glucose, maltooligosaccharide or glucan. The length of the acceptor molecule can influence the kinetic parameters of the reaction (see main text). Cyclization: an intramolecular reaction in which the glucoside produced in the hydrolytic step makes an internal glucosidic bond between its reducing end and its free 4′-position. Coupling: a cycloamylose molecule is firstly linearized in the course of the hydrolytic step and then transferred to an acceptor α-1,4′-d-glucan. Hydrolysis: only the hydrolysis of a glucosidic bond occurs without any further binding step. Coloured hexagons represent glucose units in glucan molecules (amylose, oligosaccharides and cycloamylose); open hexagons stand for glucan-reducing ends. In the coupling reaction, any linear glucoside can serve as acceptor. Figure modified from [16].

The enzymes reported in Table 1 were all expressed as recombinant proteins and tested for their activity by applying specific assays for the different reactions catalysed by 4α-GTases [16,30]: (i) disproportionation, (ii) cyclization; (iii) coupling; and (iv) hydrolysis (Figure 1). In most cases, reactions to obtain products for possible biotechnological applications were also investigated.

### 3.1. Enzymatic Activity of Extreme Amylomaltases

The main reaction characteristics of thermophilic amylomaltases that have been studied are the optimal temperature and pH, the conditions that favor cyclization versus disproportion and the capacity to modify starch. 

#### 3.1.1. Identification of Optimal Conditions of Temperature and pH and Enzyme Thermostability

Taking advantage of the evidence that glucose molecules can be obtained by the action of amylomaltases on maltooligosaccharides [9,31], the optimal temperature for enzyme activity has been determined by incubating the enzyme with one of these substrates at different temperatures and then measuring the amount of glucose released. Using maltotriose as substrate, the optimal reaction temperature was found to be 60 °C for the *T. filiformis* amylomaltase [21] and in the 70–75 °C range for the enzymes from *T. thermophilus* AT-62 [9] and HB8 [32] and *T. brockianus* [19]. Enzymes from the hyper-thermophilic bacteria *A. aeolicus* and *P. aerophilum* exhibited maximal activities at 90 °C and 95 °C, respectively [11,15]. In the case of the *T. aquaticus* amylomaltase [17], the optimal temperature of 70 °C was evaluated by following the consumption of amylose in the presence of maltose as an acceptor (a disproportionation reaction) in the presence of the iodine reagent. The residual amount of amylose was followed by measuring the optical adsorption of the amylose-iodine complex [33]. Both approaches were also adopted in some cases to establish the optimal pH of reactions. Results indicate an optimal pH in the 5.5–6.7 range [9,11,15,19,21,32], with the exception of the *T. aquaticus* enzyme, which showed a maximal activity at pH 7.5 [17]. 

The thermal stability of the enzymes, a relevant property for industrial applications, was determined by the same assays after incubation of the enzymes for a short period at increasing temperatures. The enzymes from *T. thermophilus* AT-62, *A. aeolicus* and *T. aquaticus* showed residual activities of about 90% after incubation at 80 °C [9,15,17]. Additionally, the *P. aerophilum* enzyme showed high thermostability, being able to retain 70% of its activity after 55 min at 95 °C [11].

#### 3.1.2. Reactions with Maltooligosaccharides as Substrates

Another analysis of particular interest for understanding the natural substrates of amylomaltases and for determining specific substrate preferences has been performed using different maltooligosaccharides (G1: glucose; G2: maltose; G3: maltotriose; G4: maltotetraose; G5: maltopentaose; G6: maltohexaose; G7: maltoheptaose) as single substrates in disproportionation reactions. The results are similar but do not always overlap. What is commonly ascertained is the unreactivity of G1 (tested in a few cases only) and the low effectiveness of G2 as a substrate compared to larger oligosaccharides.

Analysis for the reactions by thin layer chromatography (TLC) showed that G4–G5 were the most effective substrates for the *T. thermophilus* enzyme [9], while G4–G7 were more effective as substrates than smaller molecules for the *T. aquaticus* and *T. brockianus* enzymes [17,19]. On the contrary, in the case of *T. filiformis*, G3 was the most effective substrate, while the effectiveness of the other substrates decreased with their length [21]. 

For the *P. aerophilum* enzyme, kinetic values indicated the maximum efficiency (*kcat*/*Km*) for G3, with decreasing values for the other substrates as their length increased [11]. For *A. aeolicus*, the highest efficiency was found for G5, with decreasing values for all the other substrates [15]. Enzyme specific activity, determined in the case of the amylomaltase of *T. brockianus*, was found to decrease from G3 to G7 [20].

#### 3.1.3. Cyclization Reactions

Thermophilic and hyper-thermophilic amylomaltases have also been investigated for the synthesis of CAs of variable length by cyclization reactions. The first evidence that amylomaltases could produce CAs was from the studies of Terada et al. [9], who demonstrated the presence of glucoamylase-resistant glucans among the products of the reaction of the *T. thermophilus* enzyme on amylose. The dimensions of the glucoamylase-resistant products, determined by HPAEC (high performance anion exchange chromatography) analysis using CAs with a DP between 22 and 31 as standards, corresponded to molecules with a DP minimum of 22. The same approach demonstrated the capacity of other extreme amylomaltases to synthesize CAs, but with different sizes, such as CAs with a DP between 16 and 50 for the enzyme of *T. aeolicus* [15], DP between 19 and 35 for the enzyme from *T. aquaticus* [17] and DP between 22 and 29 for the *T. filiformis* amylomaltase [21]. Interestingly, in the last case it was also demonstrated that reaction conditions could influence the prevailing of the cyclization or disproportionation reactions. In particular, cyclization was favored by raising the temperature to 70 °C and lowering the pH to 5. It should be noted that in some cases, the synthesis of CAs of small dimensions (DP of 5 and 7) was also described for amylomaltases from the thermophilic bacteria *T. aquaticus* (using as substrate the debranched products of amylomaize, instead of the usually used potato amylose [34] or rice amylose [35]), *D. geothermalis* and *T. scotoductus* [16]. A last note must be reported for the amylomaltase from *P. calidifontis*, as this was the only enzyme found not able to synthesize CAs [12].

### 3.2. Applications of Amylomaltases 

From an applicative point of view, the interest in amylomaltases mainly derives from their capacity to modify starch, leading both to products of interest in the food industry and to the synthesis of CAs, which find applications in the pharmaceutical and chemical fields. Hyper- and thermophilic amylomaltases are particularly suitable for these processes, in consideration of the high temperatures required for the solubilization of starch.

#### 3.2.1. Starch Modifications for Applications in Food Industry

Starch is one of the major components of human nutrition, and its susceptibility to various enzymatic processes is relevant for food properties. For example, highly digestible starch causes a rapid production of glucose in human upper guts and blood stream, with severe health consequences, such as diabetes and cardiovascular complications [36]. The production of slowly digestible starch is therefore among the priorities of food industries. Jiang et al. [26] applied a treatment with amylomaltase from *A. cellulolyticus* to modify the structure and the digestibility features of corn starch. The treatment, carried out up to 12 h of incubation, resulted in reduction of amylose percentages (but with an increase in the molecular weight) and reduction of molecular weights of amylopectin molecules. An overall increase in the number of short chains (glucose units below 13) and long chains (glucose units higher than 30) was also observed. This reorganization led to increasing levels of slowly digestible and resistant starch fractions, as indicated by measurements of released glucose. 

Another starch characteristic of relevance for the food industry is its retrogradation capacity. Starch retrogradation is the process of the re-association of amylase and amylopectin chains, which occurs when gelatinized starch (obtained by the hydration of starch granules in hot water) is cooled. During retrogradation, starch chains are not re-associated with the highly ordered structure characteristic of native grains, resulting in the formation of an insoluble gel [37,38]. The process is irreversible and gels cannot be resolubilized by heating [39]. Starch treatment with amylomaltases also affects retrogradation. It was observed that when gelatinized potato starch was incubated with amylomaltase from *T. thermophilus* the viscosity and turbidity of the slurry changed dramatically, becoming much more liquid and transparent [32]. The enzyme produced the disappearance of the amylose fraction and the formation of amylopectin molecules with a broadened side-chain composition and with no detectable increase of reducing sugars. Furthermore, the modified starch showed a surprising gelation behavior compared to that of the untreated starch. Instead of forming a partially thermo-irreversible gel due to retrogradation, the amylomaltase-treated starch formed a gel that melted almost completely after heating. Amylomaltases have been therefore employed to produce thermoreversible gels to be used as alternatives to animal gelatin. Enzymes from *P. aerophilum* [11], *P. calidifontis* [13], *T. uzoniensis* [14], *T. aquaticus* [18], and *T. filiformis* [22] have been assayed for the production of thermoreversible starch gels from different sources (potato, tapioca, corn, rice, cassava, pea), with slightly different properties related to the substrates and relative amylose and amylopectin differential compositions. 

The amylomaltase from *T. thermophilus* (O87172) was also successfully used for the synthesis of glycogen, to be used in industrial applications in the food, chemical and pharmaceutical fields [40].

In light of the possible applications in the food industry, extreme amylomaltases were also produced using GRAS (Generally Recognized as Safe) organisms, such as *B. subtilis* and *Saccharomyces*, as in the cases of enzymes from *T. aquaticus* [41], *T. thermophilus* [24] and *T. filiformis* [22].

#### 3.2.2. Synthesis of CAs for Applicative Purposes

CAs are highly water-soluble molecules with a large hydrophobic internal channel, capable of hosting large organic compounds [39]. For these characteristics, they have been used as carriers of several molecules of biological interest, such as nucleic acids [42,43,44], phenolic compounds [45,46], flurbiprofen [47,48], resveratrol [49] and also as artificial chaperones for protein refolding [50]. This has, overall, raised the interest of the pharmaceutical and chemical industries. Even if CAs have been produced by employing several 4α-GTases, here we summarize only findings related to extreme amylomaltases.

In particular, CAs with DPs starting from 22 and obtained by *T. aquatics* amylomaltase from synthetic amylose exhibited chaperone properties toward chemically denaturated citrate synthase, carbonic anhydrase B and lysozyme [50]. CAs worked by effectively accommodating detergent molecules, which prevent the aggregation of chemically denatured enzymes, and by promoting proper protein folding. The study employed CAs kindly provided by Ezaki Glico Co., Ltd. (Osaka, Japan), with the specification that the enzyme was prepared by the previously reported procedure for the *T. aquaticus* enzyme [9].

A cationic CA derivative was synthesized by introducing spermine groups into a CA with DP = ∼100 and used as vector for the delivery of the luciferase gene. By using COS-7 cells, a greater transfection efficiency of the CA-spermine-DNA complex than the complex prepared without the amine was demonstrated [42]. More recently, CA cationic nanometer-sized gels (nanogels) consisting of CAs modified with cholesterol and diethylaminoethane were used to produce complexes with CpG oligodeoxynucleotides; these complexes were found effective in inducing cytokine production after delivery to macrophage-like cells [43]. A similar cholesterol-spermine based CA nanogel complex was also used to deliver the vascular endothelial growth factor (VEGF)-specific short interfering RNA into tumor cells, resulting effectively in the suppression of neovascularization and growth of renal cell carcinomas in mice [44]. It must be underlined that the synthesis of CAs by thermophilic amylomaltases was not specified in the last three studies. Nevertheless, in all cases, the employment of CAs kindly provided by Ezaki Glico Co., Ltd. (Osaka, Japan) was reported, similarly to what previously was indicated in the study of Machida et al. [50] regarding the use of CAs as chaperons.

The capacity of CAs (DP between 23 and 45), obtained from amylose by the action of *T. aquaticus* amylomaltase, to solubilize phenolic compounds was found to be effective to improve the stability of phenolic compounds of fruits and vegetables against oxidation and browning enzymes [45,46]. 

## 4. Structural Analyses

4α-GTases of the GH77 family belong to the large super-family of α-amylases, enzymes characterized by a central (β/α)_8_ catalytic domain (usually referred to as domain A) and sharing main structural features with families GH13 and GH70, which also belong to α-amylases.

Amylomaltases contains several insertions between elements of the central (β/α)_8_ domain, which are referred to as subdomains B1–B3 [51]. Four regions are conserved in the domain A of amylomaltases, corresponding to amino acidic positions 206–218, 287–297, 335–343 and 386–395 of the *T. thermophilus* sequence O87172 [51] (see below for comparisons with other amylomaltases). Another structural feature of amylomaltases is the so-called 250s loop, a region made up of amino acids around position 250 (247–255 in the *T. thermophilus* enzyme O87172) partially covering the active site cleft [51]. Another feature also shared among amylomaltases is the 460s loop (formed by amino acids in position 458–472 in the *T. thermophilus* enzyme) which, together with other amino acids (e.g., Tyr54) form the secondary substrate binding site [51].

**Figure 2:**
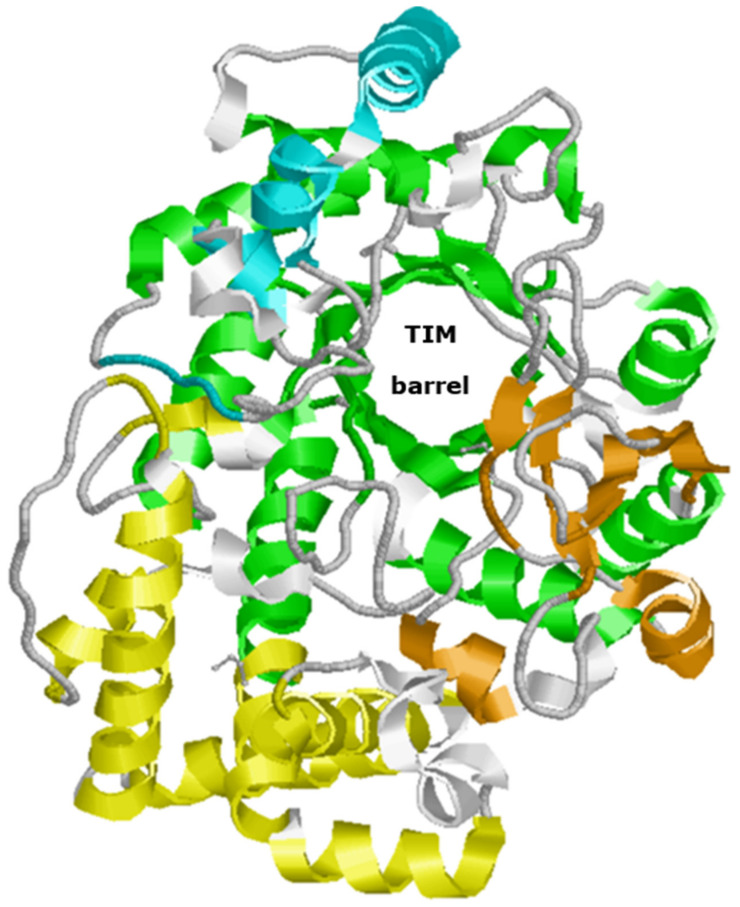
3D structure of *T. thermophilus* amylomaltase. The 1ESW structure was downloaded from PDB and coloured using RasMol [52]. Green elements correspond to the domain A (TIM-barrel). The other coloured elements correspond to subdomain B1 (orange), subdomain B2 (yellow), and subdomain B3 (cyan). Domains and colours are as reported by Przylas et al. [51]. The main functional elements (active site, 250s loop, secondary substrate binding site) are further detailed in Section 4.2.

The 3D structures of amylomaltases have been established only for a limited number of enzymes. According to the CAZy GH77 page, currently the 3D structures have been established for the *Bacteria A. aeolicus* (PDB: TZ7), *C. glutamicum* (PDB: 5B68), *E. coli* (PDB: 4S3P), *S. agalactiae* [6M6T], *T. aquaticus* (PDB: 1ESW), *T. brockianus* (PDB: 2X1I) and *T. thermophilus* (1FP8). Figure 2 shows the 3D structure of the *T. thermophilus* amylomaltase O87172, coloured according to the different domains. Other details on the structural elements of the protein are reported in Section 4.1.

### 4.1. Structural Features

According to a detailed comparison of sequences of 4α-GTases of the GH77 family [53], four distinct groups of enzymes can be distinguished: (i) prokaryotic amylomaltases of the *T. thermophilus* (O87172) type; (ii) amylomaltases from *borreliae*; (iii) plant D-enzymes and (iv) bacterial amylomaltases with a longer *N*-terminus of the *E. coli* type. Most of the enzymes reported in Table 1 have between 468 and 503 amino acids and can be assigned to the first group of enzymes. Two sequences (the archaeal MZ422727 and the bacterial A0LVB3) have peculiar extra-regions. While the bacterial enzyme can be classified in the fourth group of GH77 enzymes, no clear assignment to any of the four groups is possible for the archaeal enzyme. 

The sequence alignments of the extreme amylomaltases are shown below separately for the three groups, as obtained by the Clustal program [54]. 

#### 4.1.1. Amylomaltases of the *T. thermophilus* (O87172) Type

**Figure 3:**
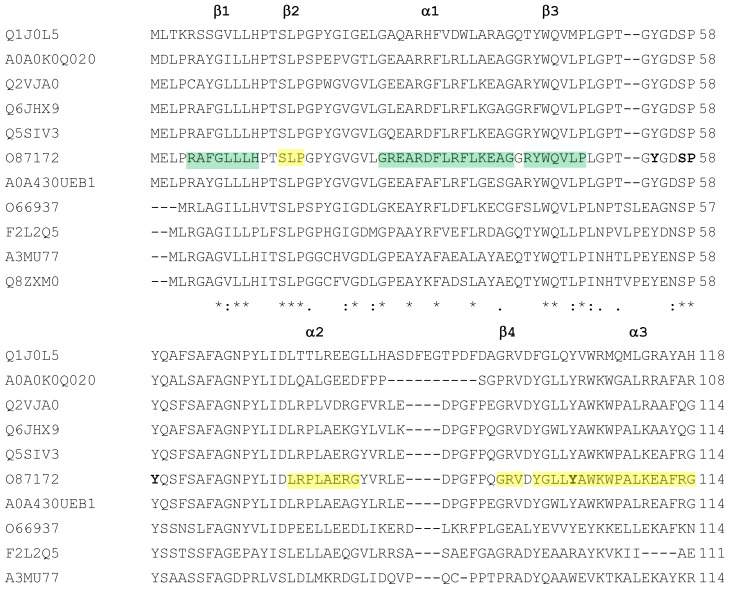

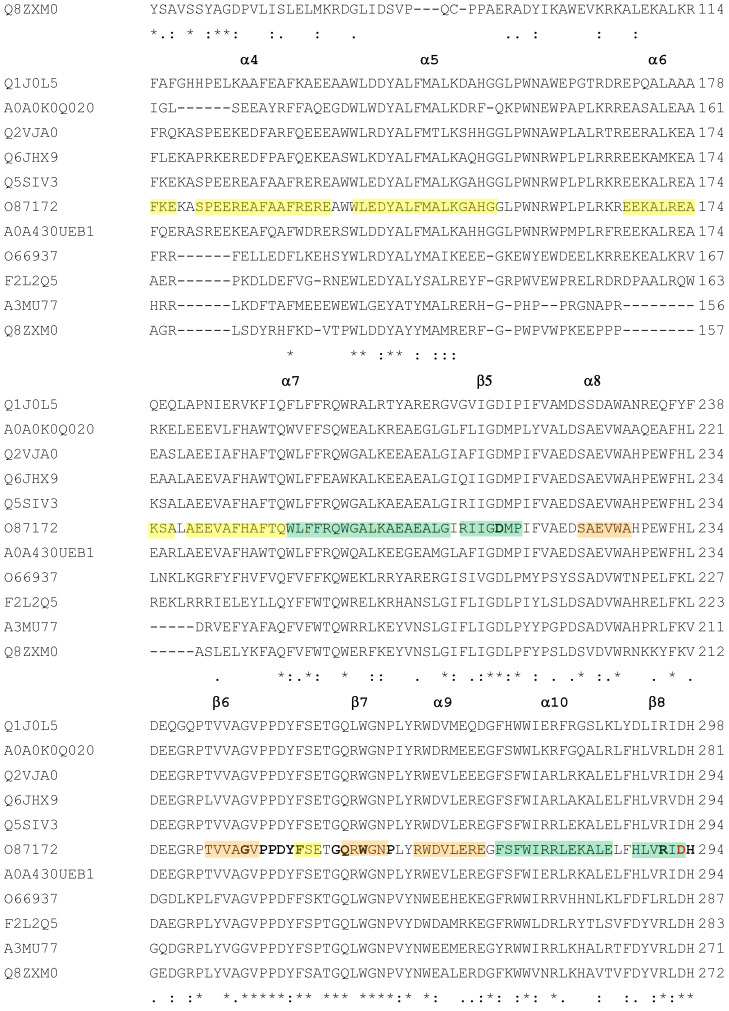

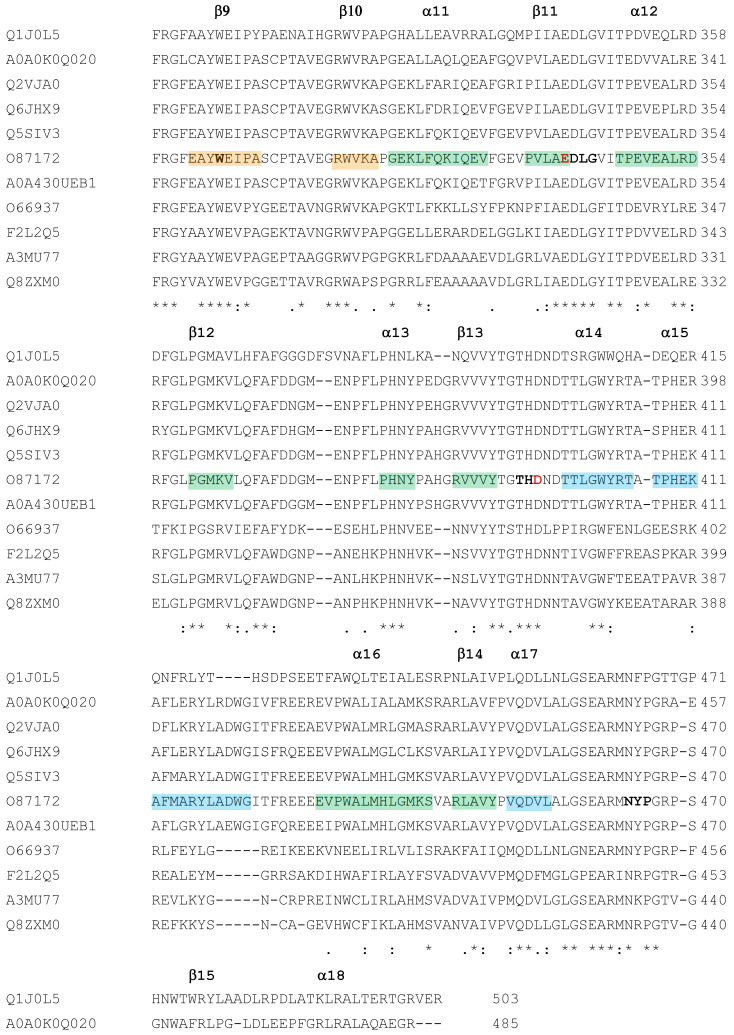

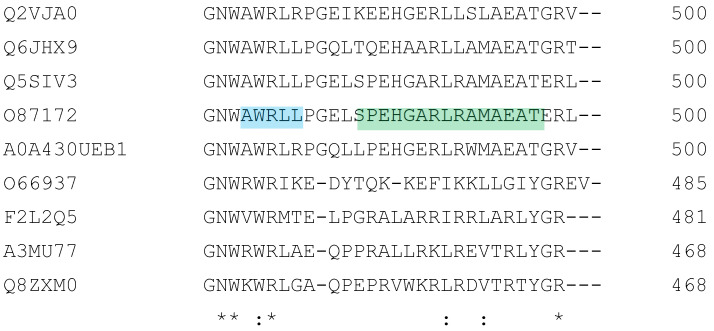
Multialignment of amylomaltases from thermophilic and hyper-thermophilic *Bacteria* and *Archaea*. Sequences of enzymes are indicated by their UniProt accession number (see Table 1). The order of the sequences and amino acid conservation symbols are as obtained by the Clustal program [54] (in particular, asterisks indicate identical residues; colons and dots indicate conserved and semi-conserved substitutions, respectively). The first eight sequences are from *Bacteria*; the other three sequences belong to *Archaea*. The secondary structure description is related to amylomaltase from *T. thermophilus* (O87172) according to the 3D structure described by Przylas et al. [51]. In particular, green regions refer to the (β/α)_8_ TIM barrel (domain A); the other coloured regions correspond to subdomain B2 (yellow), subdomain B1 (orange) and subdomain B3 (cyan). Conserved amino acids discussed in the main text are reported in bold, with the exception of the three catalytic amino acids, which are reported in red.

The alignment of the sequences of the first group is reported in Figure 3. The alignment and the identity matrix (Table S1) clearly distinguish the bacterial sequences from the archaeal counterparts. Higher identity values can be observed among sequences from the same domain (Table S1). In particular, while bacterial sequences show a large range (44.61–88.00%) of relative identity values (in this comparison only the O87172 sequence of the two almost identical sequences from *T. thermophilus* was considered), archaeal sequences show lower identity values, in the 58.84–75.11% range. In the comparison between archaeal and bacterial sequences, identity percentage never goes above 48.62. It is interesting to underline the case of the sequence from the hyper-thermophilic bacterium *A. aeolicus* (O66937). It has the lowest identity values with the other bacterial sequences (44.61–46.67%), supporting the hypothesis, based on phylogenetic analysis of 16S ribosomal RNA sequences ([6] and references therein), of *Aquificaceae* as one of the most deeply branching family within the bacterial domain. Not considering the *A. aeolicus* sequence, the range of identities among bacterial amylomaltases would rise to 53.65–88.00%.

Specific insertions/deletions differ between bacterial and archaeal sequences, but never affect regions known to be involved in the formation of the (β/α)_8_ TIM barrel. Almost all amino acids involved in the formation of the active site and interactions with substrates and products [51] are fully conserved. This includes, in particular:

- The three catalytic amino acids of the active site (D293, E340 and D395, according to the O87172 sequence from *T. thermophilus*), together with those generally conserved in the active site of amylomaltases (Y59, D213, R291 and H394) and other amino acids that form the core of the catalytic cleft (W258, H294, L342, N464) or are part of it (S57, P58, D341, G343, T393 and P466);
- The amino acids with solvent-exposed hydrophobic side chains (Y250, F251, Y54, Y101 and Y465) which are supposed to interact with the hydrophobic face of CAs. One exception is Y465 in amylomaltases from *Archaea*, which is substituted by amino acids with positively charged side chains (R and K);
- Amino acids of the so-called 250s-loop, thought to be involved in the amylomaltase-specific synthesis of CAs, (P247, P248, D249, G255) and some of the flanking β6 and β7 strands (G245, Q256, W258, P261, W302).

#### 4.1.2. Amylomaltases of the *E. coli* Type

**Figure 4:**
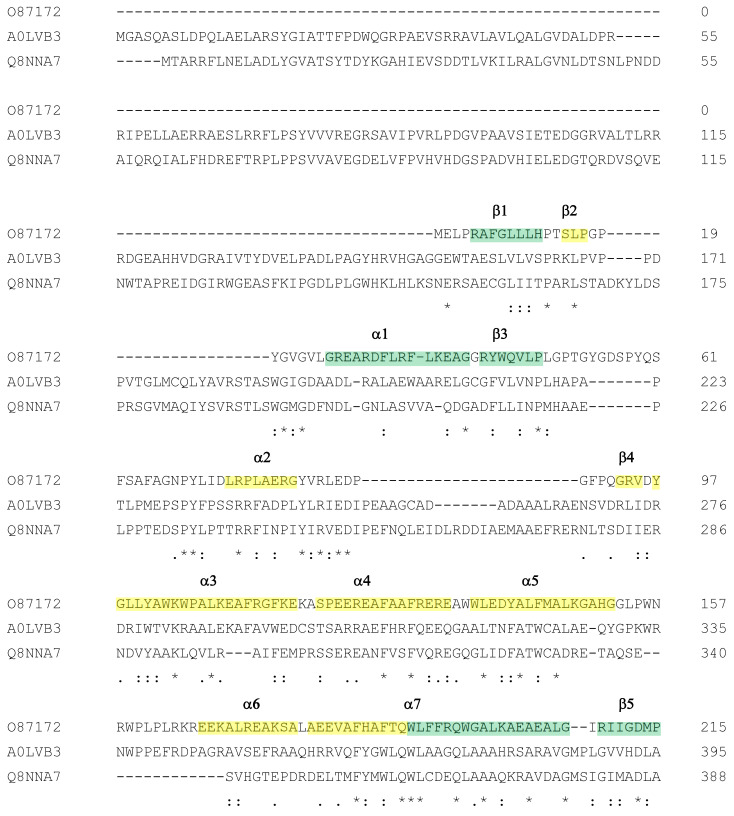

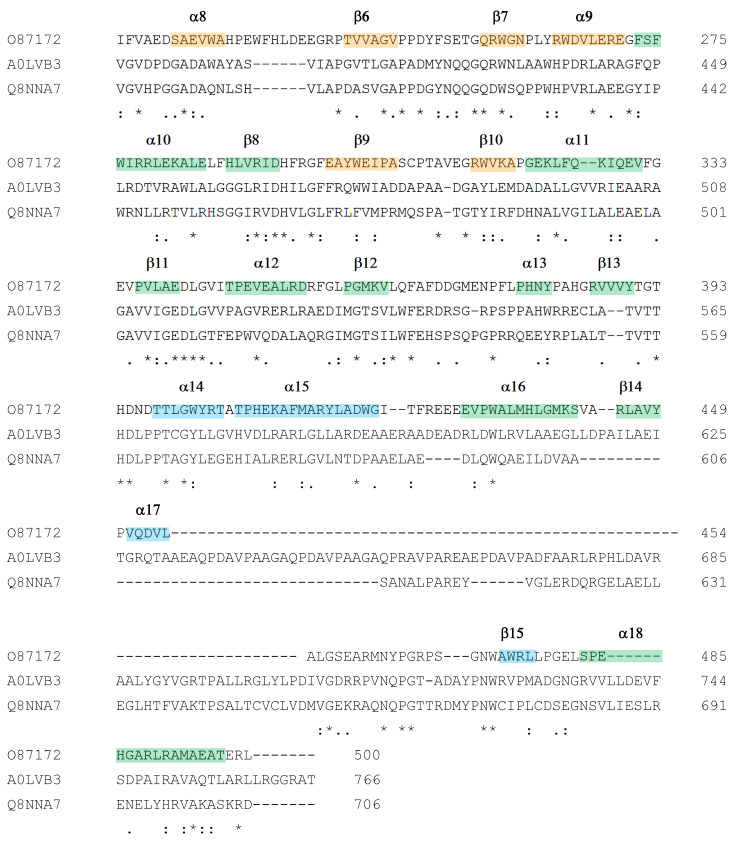
Multialignment of amylomaltases from *A. cellulolyticus* (A0LVB3), *C. glutamicum* (Q8NNA7) and *T. thermophilus* (O87172). Sequences of enzymes are indicated by their UniProt accession number (see Table 1). The order of the sequences and amino acids conservation symbols are as obtained by the Clustal program [54] (in particular, asterisks indicate identical residues; colons and dots indicate conserved and semi-conserved substitutions, respectively). Secondary structure description is related to amylomaltase from *T. thermophilus* according to the 3D structure described by Przylas et al. [51]. In particular, green regions refer to the (β/α)_8_ TIM barrel (domain A); the other coloured regions correspond to subdomain B1 (orange), subdomain B2 (yellow) and subdomain B3 (cyan).

Among the extreme amylomaltases reported in Table 1, the *A. cellulolyticus* enzyme (A0LVB3) is about 250–300 amino acids longer than the other enzymes and could belong to the group of amylomaltases with a longer *N*-terminus of the *E. coli* type [53]. The recombinant enzyme was found effective in enhancing the slowly digestible and resistant fractions of corn starch, but its structural features were not analysed [26]. Other amylomaltases of similar lengths have already been described in *Bacteria*, including *E. coli* [55]. They are characterized by the presence of a *N*-domain of about 160 amino acids in front of the TIM-barrel catalytic domain. Phyre2 analysis showed that the *A. cellulolyticus* sequence can be folded in a 3D model with 100% confidence with the structure of the amylomaltase from the bacterium *C. glutamicum* [7]. The percentage of identity of the two sequences is about 42%. A possible role of the *N*-terminal domain could be that of a carbohydrate-binding module (CBM) as the presence of an immunoglobulin-like β-sandwich fold would suggest [7]. Currently, the *A. cellulolyticus* enzyme is the only extreme amylomaltase possessing the *N*-terminal domain to have been expressed as a recombinant protein and functionally tested. Figure 4 reports the multialignment of the amylomaltases of *A. cellulolyticus* and *C. glutamicum* together with that of the more compact amylomaltase from *T. thermophilus* (O87172) for comparison. The alignment shows that the two longer enzymes have several extra-regions compared to the structure of the short enzyme. The longest extra sequence of about 150 amino acids is located at the *N*-terminus. Moreover, there are other insertions and deletions in the subdomain B2, one deletion in the subdomain B1 and another large insertion in the subdomain B3. The lengths of the structural elements of the TIM-barrel are more conserved.

Amino acids involved in the formation of the active site and interactions with substrates and products are mostly conserved, also in comparison with amylomaltases of the *T. thermophilus* group. Deviations are related to a deletion of seven amino acids in the sequence of the *A. cellulolyticus* enzyme corresponding to amino acids 54–60 of the *T. thermophilus* sequence, which determines the absence of the amino acids 57SPY59, generally conserved in the amylomaltases of the first group. The same deletion is present in the mesophilic enzyme from *C. glutamicum*, taken as comparison for amylomaltases with a longer *N*-terminus. Another deviation in the active site domain can be detected in correspondence of F217 of the *T. thermophilus* enzyme. In this position, a Phe or a Tyr residue can be identified in hyper- and thermophilic enzymes of the *T. thermophilus* group (Figure 3), while in the amylomaltases with longer *N*-terminus (both of the thermophilic and mesophilic type) a Gly is present (Figure 4). Other interesting deviations can be observed in correspondence of the Pro residues 248 and 261 of the 250s loop of the *T. thermophilus* enzyme. These amino acids are present in all the extremozymes of the first group (Figure 3) and might indicate a strong requirement for a rigid structure. Nevertheless, both the residues are substituted by Ala (A422 and A435) in the enzyme from the thermophilic *A. cellulolyticus*, while two Pro residues can be found in the mesophilic counterpart from *C. glutamicum* (Figure 4). Other differences among the amylomaltases of the two groups are summarized in Table S2.

#### 4.1.3. Amylomaltases Not Yet Classified

Another extreme amylomaltase with peculiar extrasequences is that from the halophilic *Archaea H. walsbyi* (Table 1). Its gene was detected in the course of a functional metagenomic analysis of the saltern of Margherita di Savoia, located on the south-eastern coast of Italy, and the characterization of the recombinant enzyme is in progress (Ceci, LR, manuscript in preparation). Assignment of the sequence to the *H. walsbyi* species was possible on the basis of the perfect identity of the gene sequence with genomic sequences of *H. walsbyi* species (NBCI acc. numbers FR746099.1 and AM180088.1) as indicated by BLAST analysis (not shown). The *Haloquadratum* genus was found in the 13.1–36.0% salinity range of the saltern of Margherita di Savoia, with the highest relative abundance (about 63%) in a pond with 36.0% salinity [56].

**Figure 5:**
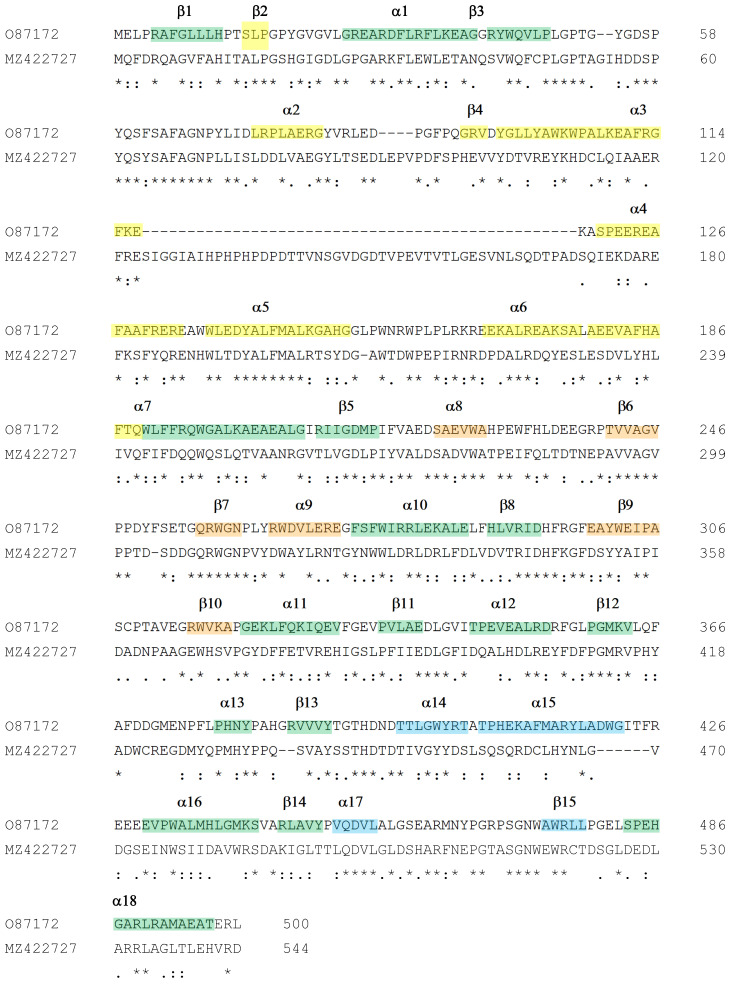
Multialignment of amylomaltases from *H. walsbyi* (MZ422727) and *T. thermophilus* (O87172). Sequences of enzymes are indicated by the UniProt accession number for the *T. thermophilus* enzyme and by the GenBank accession number for the *H. walsby* enzyme. Other details of the sequences are reported in Table 1. The order of the sequences and amino acid conservation symbols are as obtained by the Clustal program [54] (in particular, asterisks indicate identical residues; colons and dots indicate conserved and semi-conserved substitutions, respectively). Secondary structure description is related to amylomaltase from *T. thermophilus* according to the 3D structure described by Przylas et al. [51]. In particular, green regions refer to the (β/α)_8_ TIM barrel (domain A); the other coloured regions correspond to subdomain B1 (orange), subdomain B2 (yellow) and subdomain B3 (cyan).

Blast analysis of the *Haloquadratum* amylomaltase showed that its extrasequence is peculiar to halophilic *Archaea* and not shared by thermophilic microorganisms (not shown). Figure 5 shows the alignment of the amylomaltases from *H. walsbyi* and *T. thermophilus* together with the description of the secondary structures from *T. thermophilus* as reported by Przylas et al. [51]. The main structural difference suggested by this comparison is a large region of about 50 amino acids located between the α3 and α4 helices of the subdomain B2. Other differences are detailed and discussed in the Section 4.2.

### 4.2. Enzyme Conserved Regions

**Figure 6:**
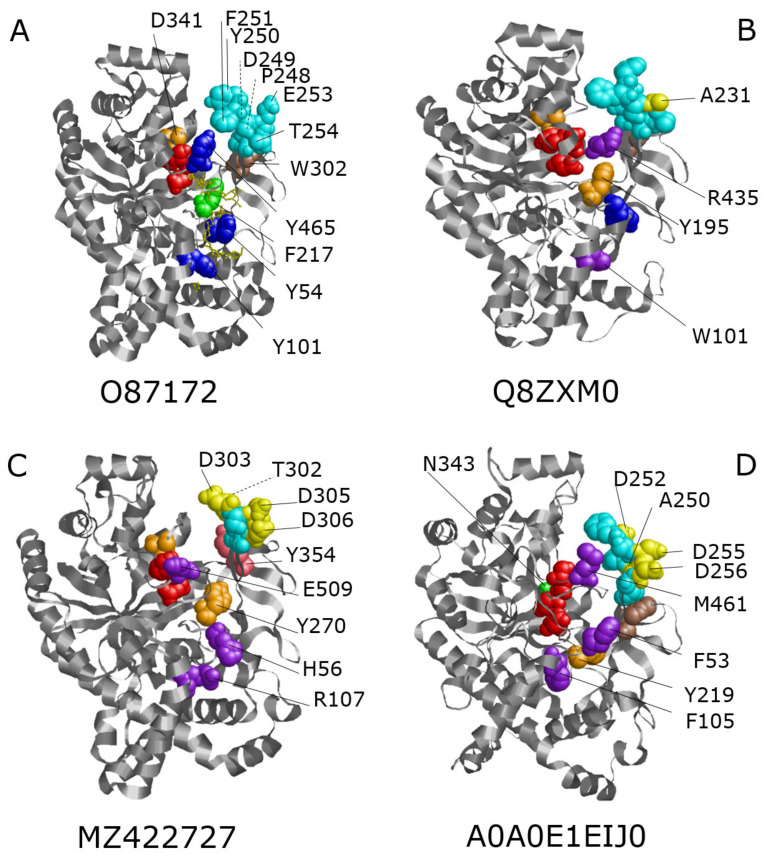
3D structures of selected amylomaltases and amino acids of conserved regions. The reported structures refer to amylomaltases O87172 from *T. thermophilus* (**A**), Q8ZXM0 from *P. aerophilum* (**B**), MZ422727 from *H. walsbyi* (**C**) and A0A0E1EIJ0 from *S. agalactiae* (**D**). See also Table S2 for amino acids positions. The dashed lines indicate amino acids located at the rear of the structure. Coloured spacefillings are explained in the text. In the structure of the *T. thermophilus* enzyme, molecules of acarbose (a maltotetraose derivative, acting as an inhibitor of enzymes of the a-amylase family and usually used for studying possible binding modes for large amylose substrates [57]) are also shown.

We also examined three-dimensional structural details of conserved regions/amino acids in some representative amylomaltases in order to highlight possible characteristics related to their different reaction conditions. The selected enzymes were the archaeal hyper-thermophilic enzyme Q8ZXM0 from *P. aerophilum*, the archaeal halophilic enzyme MZ422727 from *H. walsbyi*, the bacterial thermophilic enzyme 087172 from *T. thermophilus* and the bacterial mesophilic enzyme A0A0E1EIJ0 from *S. agalactiae* (selected regions and amino acids are reported in Table S2). The bacterial enzymes were chosen for the availability of their 3D structures; the archaeal hyper-thermophilic enzyme was chosen among the three available enzymes because it is the one with the lowest similarities with the bacterial counterparts. The *H. walsbyi* amylomaltase was selected as the only halophilic enzyme (see Table 1). For the enzymes for which the 3D structures were not available, the Phyre2 structural models were used. Definitions and lengths of conserved regions and numbering of amino acids refer to the *T. thermophilus* 087172 sequence [51], when not differently indicated. Specific amino acid differences were highlighted by coloured spacefilling representation of the 3D structures of the four enzymes (Figure 6) as described below for the (i) active site, (ii) the 250s loop and (iii) the secondary substrate binding site.

#### 4.2.1. Active Site

The active site is highly conserved in amylomaltases. It contains the three catalytic residues (D293, E340 and D395, shown as red spacefilling in Figure 6A–D) and the other amino acids which form the catalytic cleft around the active center (see Table S2). One exception is the acidic residue D341, which is conserved in all the extremozymes (Figure 2, Figure 3 and Figure 4) but is substituted by the polar N343 in the mesophilic enzyme A0A0E1EIJ0. D341 in O87172 and the corresponding residues in Q8ZXM0 and MZ422727 (D319 and D393, respectively) are reported in orange in Figure 6A–C, while the variation N343 in the mesophilic enzyme is reported in green in Figure 6D.

Another difference occurs in the bacterial thermophilic enzyme O87172, which presents the aromatic amino acid F217 (in green in Figure 6A) instead of the otherwise conserved Tyr (residues Y195, Y270 and Y219 in Q8ZXM0, MZ422727 and A0A0E1EIJ0, respectively, coloured in orange in Figure 6B–D). Among hyper-thermophilic and thermophilic amylomaltases, this position is about equally occupied by either Phe or Tyr, but archaeal enzymes have only Tyr (Figure 2). α-amylases show a strongly conserved His in this position [51], leading to hypotheses regarding the requirement of aromatic amino acids in the specific position.

#### 4.2.2. 250s Loop

The 250s loop region (corresponding to residues P247-G255 in the bacterial thermophilic enzyme O87172 and coloured in cyan in Figure 6A–D) is generally conserved among amylomaltases (see also Figure 2, Figure 3 and Figure 4 and Table S2). Nevertheless, some interesting variations can be observed. While P248 is conserved in all the extremozymes, an A250 is present in the mesophilic *S. agalactiae* enzyme A0A0E1EIJ0 (shown in yellow in Figure 6D). The Pro residues in the extremozymes seem important to bend the lid structure of the 250s loop on the active site. The Ala residue in the mesophilic structure might be related to a higher flexibility in receiving the substrate.

Some changes can also be observed in the sequences forming the tip of the loop, which might regulate access to the active site. In correspondence of the sequence 249DYF251 in the thermophilic enzyme O87172, the halophilic enzyme MZ422727 shows the variations 302TD(-)303, while the mesophilic enzyme A0A0E1EIJ0 shows a variation in correspondence of Y250, substituted by D252 (both changes are coloured in yellow in Figure 6C,D, respectively).

The acidic residue E253 is substituted by Asp in both the halophilic and mesophilic enzymes (residues D305 and D255, respectively, shown in yellow in Figure 6C,D), while a neutral Ala is present in the hyper-thermophilic enzyme (A231 in Figure 6B). 

A last difference can be observed in the correspondence of T254, which is substituted by acidic residues in the halophilic and mesophilic enzymes (residues D306 and D256, respectively, coloured in yellow in Figure 6C,D). These variations are currently not easy to interpret, as they increase the surface charges in two enzymes with different reaction conditions.

In this region, we decided to include also W302 (shown as brown spacefilling in Figure 6A,B,D), since it is supposed to support the 250s loop [51]. The amino acid is fully conserved among thermophilic amylomaltases (Figure 2) but not in the archaeal halophilic enzyme (MZ422727), in which it is substituted by a more polar Tyr (residue Y354, shown in pink in Figure 6C).

#### 4.2.3. Secondary Substrate Binding Site

A lower degree of conservation can be observed in this site. The solvent-exposed residues Y54, Y101 and Y465 of the bacterial thermophilic enzyme O87172 (shown in blue in Figure 6A) are located along an alternative glucan binding groove near to the catalytic cleft [51]. The archaeal enzyme Q8ZXM0 maintains the first Tyr (Y54 in blue in Figure 6B), while the other two Tyr residues are substituted by Trp and Arg, respectively (W101 and R435, in purple in Figure 6B). Changes are more drastic in the halophilic enzyme, being the three positions occupied by His, Arg and Glu, respectively (H56, R107 and E509 in purple in Figure 6C). In the mesophilic enzyme A0A0E1EIJ0, other amino acids are present in the three positions (F53, F105 and M461), which maintain the hydrophobic character detectable in the bacterial extremophile counterpart (shown in purple in Figure 6D). In conclusion, only the hydrophobic nature of the first residue (Y54) is conserved among the four amylomaltases. The two bacterial sequences share the hydrophobicity of the other two residues, while archaeal hyper-thermophilic and halophilic sequences show the presence of one and two charged amino acids, respectively.

Of interest is the analysis of the amino acid sequence around Y54, characterized by the presence of several Gly (48PLGPTGYGDSP58). This sequence is highly conserved in the bacterial short thermophilic enzymes but not in the archaeal counterparts or in the bacterial long enzymes (Figure 2 and Figure 3). One exception among the short enzymes is, once again, the bacterial amylomaltase from *A. aeolicus* (066937), whose sequence for this trait shows higher similarities (between 54 and 69%) with the archaeal amylomaltases than with the bacterial counterparts. These differences would imply different interactions with the substrate of the bacterial and archaeal enzymes.

### 4.3. Analysis of the Amino Acid Composition and Associated Structural Features

In order to illustrate possible correlations between the amino acid content of extreme amylomaltases and their activities in extreme conditions, the following criteria were considered: amino acid composition and isoelectric point (IP), hydrogen bonds (HBs) and salt bonds (SBs).

#### 4.3.1. Amino Acid Composition and IP

Percentages of amino acid composition and IPs of the amylomaltases reported in Table 1 were computed using the ProtParam tool available in Expasy [58] (Table 2). 

Taking into consideration only the values reported for the enzymes of the *T. thermophilus* type, it appears that only for a few amino acids a clear trend can be observed which distinguishes the enzymes from hyper- and thermophiles from the mesophilic enzymes. This is the case of Arg, which shows a consistently higher percentage in thermophilic enzymes than in the mesophilic counterpart, or Leu, which has its highest percentage in the mesophilic enzyme. Interestingly, only in a few cases, the reported variations of the amino acid compositions between thermophilic and mesophilic amylomaltases are in agreement with the variations described in the course of an extensive analysis of 2194 enzyme families from mesophilic and thermophilic bacteria [59]. In the course of this analysis, a subset of 1005 protein families was identified which showed, according to variations in the optimal growth temperature of the different bacteria, not only specific preferences in the types of amino acids, but also changes in the identities and frequencies of site-specific residues, and in the identities and frequencies of physically interacting temperature-associated residue pairs and variations of some networks of temperature-associated residues. According to this analysis, the amino acid Ile is characterized by a strong increase in frequency in the enzymes of bacteria with a high optimal growth temperature, while the other two branched-chain amino acids, Leu and Val, often also considered necessary to thermophilic enzymes to increase internal hydrophobic interactions [60], show no significant changes. Differently, in amylomaltases, Leu and Val have higher percentages in thermophilic enzymes, while Ile shows its higher percentage in the mesophilic enzyme (see Table 2). It should be noted, however, that for most of the thermophilic amylomaltases, the overall percentage for the three amino acids is higher than that for the mesophilic counterpart. Exceptions occur for the enzymes of *D. geothermalis* and *T. scotoductus*.

In amylomaltases, the percentages of the charged amino acids Arg, Lys, Asp and Glu, considered important for protein thermostability as they can increase the number of salt bridges and hydrogen bonds [60], only partially match the results of the analysis of the 1005 enzyme families. In fact, while the analysis of the 1005 families indicated a strong association only of Lys and Glu and thermophilic enzymes, in amylomaltases, a positive association with thermophilic enzymes can be observed for Arg and Glu (this observation can also be extended to the archaeal enzymes). Lys and Asp are always less abundant in all the thermophilic amylomaltases than in their mesophilic counterparts, with the only exception limited to Lys in the enzyme from the hyper-thermophilic bacterium *A. aeolics*.

It should be noted, however, that the extensive association analysis of amino acid percentages and enzyme thermostability conducted among 2194 bacterial enzyme families did not identify a common molecular mechanism underlying protein thermostability. Other paths, in addition to those described for the subset of 1005 families, are expected to be possible, which might better fit with the observations obtained for amylomaltases.

The enzyme from the halophile *H. walsbyi* (MZ422727) shows a particular abundance of Asp residues compared to other enzymes, together with the lowest value of Glu. Overall, it is the enzyme with the highest percentage of amino acids with acidic residues and with the lowest percentage of positively charged amino acids (Lys and Arg). 

The IP shows some differences between the different categories of enzymes, although a clear meaning cannot be assigned. While thermophilic and hyper-thermophilic enzymes from *Archaea* have the highest IP values (7.25–8.46), those from *Bacteria* have slightly acidic values (5.11–5.72). The only bacterial hyper-thermophilic enzyme, detected in *A. aeolicus*, differs with an out-of-range value (6.30) among *Bacteria*. The bacterial mesophilic enzymes have lower IPs than their thermophilic counterparts (4.75–4.80) and, finally, the sole halophilic enzyme (from *H. walsbyi*) shows the lowest IP of all the enzymes. Hence, it would appear that thermal stability and IP values have the same trend. The tendency for high IP values would be even greater in archaeal enzymes.

#### 4.3.2. Intrachain Interactions

The possibility of amino acids to establish HBs and SBs in amylomaltases has been evaluated by specific tools available at the PIC (Protein Interactions Calculator) site [61] based on the analysis of 3D protein structures. For enzymes for which the 3D structure was not available, their highest confidence Phyre2 model (Table 1) was used. The numbers of predicted HBs (distinguished in those among atoms of the main chain, HB-M, and those for atoms of the side chains, HB-S) and SBs are reported in Table 2. Some differences can be recognized among enzymes. A clear difference concerns the archaeal thermophilic and hyper-thermophilic enzymes and their bacterial counterparts, even if (once again) the bacterial sequence from *A. aeolics* diverges from the other bacterial counterparts. In comparison with the mesophilic enzyme (A0A0E1EIJ0 from *S. agalactiae*), while the bacterial thermophilic enzymes show a higher number of HBs (usually considered as a strategy to obtain a higher stability at high temperatures), the same does not happen for the archaeal enzymes. This can be observed both for HB-Ms (ranging between 161 and 165 in *Archaea* and between 225 and 249 in *Bacteria*) and for HB-Ss (included in the 5–10 and 14–30 ranges, respectively). For the archaeal amylomaltases and for the unusual bacterial enzyme from *A. aeolics*, probably other structural properties contribute to the thermostability of the enzymes. Fewer HBs than its mesophilic counterpart are also observed for the long-type amylomaltase (Q8NNA7 from *A. cellulolyticus*).

The archaeal halophilic amylomaltase has an exceptionally (for an archaeal enzyme) high number of HB-Ms (241) and no HB-S. In comparison with the other archaeal enzymes, it shows the lowest cumulative percentage of hydrophobic amino acids (Phe, Met, Trp, Gly, Ala, Val, Leu, Ile, Pro) of 48.4% versus 53.9–55.1% and the highest percentage of polar amino acids (Cys, Asn, Thr, Ser, Tyr, Gln) of 24.7% versus 16.5–19.6%. This leads to the hypothesis for this enzyme of a very compact structure with several charged and polar residues exposed on the surface. This kind of arrangement is quite common for enzymes from archaeal halophiles due to the high saline concentrations of their cellular environment [62].

The situation is more variable in the comparison of salt bridges. Although the effects of salt bridges on the thermostability of proteins have been variously evaluated, it has been found that a large number of salt bridges are present in different families of thermostable proteins [63]. In the case of amylomaltases, however, it does not occur. Bacterial thermophilic enzymes have a number of SBs ranging between 10 and 19 (for the short type) and nine for the long type. Mesophilic counterparts in Table 2 have 18 and 26 SBs. Archaeal proteins show a lower number of SBs, going from six to eight.

## 5. Mutational Analyses

A few mutational studies have been carried out on extreme amylomaltases in order to investigate the role of specific amino acids. In some cases, the possibility of favouring one specific reaction (e.g., cyclization) over the other possible reactions has also been verified by site-directed mutagenesis. In addition, we report here some mutational experiments carried out on mesophilic amylomaltases with the aim to increase their thermal stability.

The effect of the substitution of Y54 in the *T. thermophilus* amylomaltase 081872 in promoting the cyclization reaction instead of simple hydrolysis was investigated by saturated mutagenesis [64]. Several mutants (Y54A, Y54D, Y54G, Y54I, Y54L, Y54N, Y54P, Y54S, Y54T, Y54V and Y54W) showed a more than four-fold reduction in the hydrolytic activity/cyclization activity ratio. 

Site-directed mutagenesis in *T. thermophilus* HB8 was carried out to study the role of D249 and F366 in substrate binding [65]. These amino acids were predicted to interact with the substrate in putative acceptor subsites +2 and +3. The D249S mutation at subsite +2 resulted in a very low hydrolytic activity on potato starch, which prevented any further characterization of the mutant, but confirmed the relevance of Asp249 for the catalysis. The F366L substitution in subsite +3 had a less severe effect on the enzyme activity, resulting in a mutant with an activity comparable to that of the wild type enzyme. 

Mutations F251G in the 250s loop and the adjacent Q256G and W258G were studied in *T. brockianus* [20]. Generally, these mutations reduced the specific activity of the enzymes (measured against maltotriose and amylose) and also reduced *K*_m_ and *k*_cat_/*K*_m_, calculated using maltotriose as substrate. The Q256G mutation had no practical effects on the hydrolytic activity/cyclization activity ratio, while the other two mutations reduced the cyclization activity.

The E27R-mutated *T. filiformis* amylomaltase showed a significant increase in stability at a higher pH and temperature than the wild type [21]. This position in bacterial amylomaltases is not conserved, being occupied by either neutral, polar or charged amino acids (Figure 2). The mutation was introduced to increase the dimension of an Arg cluster (R27–R30–R31–R34) on the enzyme surface. It was also observed that, while the disproportionation reaction showed no change in optimum temperature and pH between the mutant and the wild type enzyme, the cyclization reaction showed a shift of the optimum pH (from pH 5.0 to 6.0) and an increase of optimal temperature from 70 to 80 °C.

More recently, a broader mutational analysis of *T. thermophilus* amylomaltase was carried out, which provided useful elements to clarify the mechanisms of substrate recognition and CA synthesis [66]. An inactivated double mutant (D293A, D395N) was used to obtain the 3D structure of the enzyme-CA complex. Although CAs with DP in the 22–45 range were used, only the molecule with 34 glucose units was found to crystallize with the enzyme. A symmetric complex consisting of two enzyme molecules sharing a single CA molecule (PDB: 5JIW) was detected. Each of the enzyme molecules of the complex is spanned by one-half of the CA, which interacts with the secondary substrate binding site, the active site and reaches the 250s loop. The observed helical structure of CA around Y54 in the secondary substrate binding site would resemble the helix of the amylose substrate in size and structure, indicating the Y54-associated pocket as the initial recognition site for polymeric substrates. In agreement with this model, the Y54G and Y101G mutants showed a reduced enzyme activity. On the basis of these results, it was also postulated that the extended crevice of the enzyme molecule could act as a molecular ruler, determining the minimal ring size for CA products of about 22 glucose units. This model, however, does not explain the identification of small-sized CAs (DP 5–7) reported for several amylomaltases, including for that of *T. thermophilus* (see [16,34,35] in Section 3). In the same study, the hypothesized role of the 250s loop in the amylomaltase transglycosylation activity was confirmed by the reduced activity of the Y250S, F251S double mutant. Furthermore, the reduced activity of the D370S mutant confirmed the hypothesis of the presence in the enzyme molecule of a succinimide dehydration product (deriving from post-translational modifications of D370 and G371), possibly involved in interactions (e.g., by H-bonds) with glucose units of the substrate.

In the cases of mesophilic amylomaltases, mutagenesis was adopted to improve the enzyme thermostability and characteristics, such as the capacity to synthesize large CAs. In the amylomaltase from the mesophilic bacterium *C. glutamicum*, the mutagenesis Y172A was performed to determine its effect in the control of cyclization reactions [67]. Overall, the mutant showed lower disproportionation, cyclization and hydrolysis activities than the wild type. At a long incubation time, the mutant showed CAs with a degree of polymerization of 28 or 29, while the principal product of the wild type enzyme was a CA of 25 glucose units. An error-prone PCR assay was also carried out to isolate enzymes with higher thermostability. It allowed for the isolation of the A406V mutant, which showed a higher thermostability at 50 °C than the wild type [68]. The catalytic efficiency values *k*_cat_/*K*_m_ of the mutant was 2.9 times higher than that of the wild type. A406V also gave higher CAs yields than the wild type. 

The mesophilic *S. agalactiae* amylomaltase was also subjected to mutation experiments [25]. In particular, C446, which is conserved in other amylomaltases from mesophiles such as *E. coli* and *C. glutamicum*, was substituted with a Pro residue, because it is highly conserved in thermophilic amylomaltases (P450 in O87172 in Figure 2) and is thought to contribute to enzyme rigidity and stability. However, more interesting results were obtained with the C446A and C464S mutants. C446A showed higher activity and higher *k*_cat_/*K*_m_ values towards the starch substrate than the wild type enzyme. The C446S mutation originated an enzyme with a 5 °C increase in optimal temperature and a three-fold increase in half-life to 45 °C, most likely due to an increase in the number of H-bonds.

## 6. Conclusions

Amylomaltases constitute an interesting research topic thanks to their capacity to modify starch (one of the most abundant compounds on Earth) in such a way as to obtain various products of great interest for biotechnological applications. Currently, however, amylomaltases investigated in extremophiles, which could allow a wide range of reaction conditions useful for industrial applications, are limited. Nevertheless, the enzymes fully confirmed their capacity to produce valuable products (CAs, starch with different retrogradation properties, thermoreversible gelatin) at high temperatures and possibly at low water activity (in the case of halophilic enzymes). Studies were also carried out to understand which structural features are required for attributing extreme capacities to these enzymes in order to introduce beneficial mutations. Some site-directed mutagenesis analyses only partially investigated the relevance of single amino acids. 

We carried out a simple comparative analysis of the most relevant sites of extreme amylomaltases, taking mesophilic counterparts for comparison. From the analysis of amino acid composition, it appears that, at least for thermophilic amylomaltases, some specific compositions might be the basis of enzyme stability. Values that slightly differentiate thermophilic enzymes (either *Archaea* or *Bacteria*) from their mesophilic counterparts are the percentages of charged amino acids. Nevertheless, in some cases the difference with the mesophilic counterpart is low and differences should be considered as indicative only. More functional validation assays are required to firmly establish the influence of specific amino acid changes on thermostability. The IP values, however, seem more strictly correlated with thermostability and with the different origins of enzymes. They are comparatively high for thermophilic archaeal enzymes, low for bacterial counterparts and even lower for mesophilic amylomaltases. As for the only halophilic enzyme included in the review, it is characterized by the absolute highest value of acidic amino acid content and the lowest number of positively charged amino acids, leading to the lowest value of IP. It also shows the highest value of HB in the main chain. The number of SBs in extreme amylomaltases, even if correlated with the different enzymes, does not allow for the establishment of simple correlations.

It is currently difficult to extrapolate general conclusions on the structure/activity relationships in extreme amylomaltases, and further studies and more enzymes to characterize are still needed. In a complementary way, the application of direct evolutionary approaches (gene-shuffling, phage-display, error-prone PCR) could further contribute to the analysis of amylomaltases and possibly provide new enzymes to study.

## Figures and Tables

**Table 1:** Amylomaltases characterized in extreme *Archaea* (green background) and *Bacteria* (cyan background).

Organism/Classification	UniProt	Length	PDB	Ref.	GH77 Group
*Pyrobaculum aerophilum*/H	Q8ZXM0	468	1TZ7_P_	[11]	1
*P. calidifontis*/H	A3MU77	468	1TZ7_P_	[12,13]	1
*Thermoproteus uzoniensis*/T	F2L2Q5	481	1TZ7_P_	[14]	1
*Haloquadratum walsbyi*/Ha	MZ422727 *	544	1ESW_P_	Unpub.	N.A.
*Aquifex aeolicus*/H	O66937	485	1TZ7	[15]	1
*Deinococcus geothermalis*/T	Q1J0L5	503	1ESW_P_	[16]	1
*Thermus aquaticus*/T	Q6JHX9	500	1ESW_P_	[17,18]	1
*T. brockianus*/T	Q2VJA0	500	2X1I	[19,20]	1
*T. filiformis*/T	A0A0K0Q020	485	1ESW_P_	[21,22]	1
*T. scotoductus*/T	A0A430UEB1	500	1ESW_P_	[23]	1
*T. thermophilus* HB8/T	Q5SIV3	500	1ESW_P_	[24]	1
*T. thermophilus AT-62*/T	O87172	500	1ESW	[9] **	1
*Streptococcus agalactiae*	A0A0E1EIJ0	498	6M6T	[25]	1
*Acidothermus cellulolyticus*/T	A0LVB3	766	5B68_P_	[26]	4
*Corynebacterium glutamicum*	Q8NNA7	706	5B68	[27,28]	4

Legend: Classification refers to hyper-thermophiles (H), thermophiles (T) and halophiles (Ha). GH77 groups are defined in the Section 4.1. Entries with a white background are related to enzymes from mesophilic *Bacteria* used for comparison in this review. * The accession number refers to the GenBank database, as the UniProt accession number is not yet available. ** Structures of covalent intermediates with acceptors were provided by Barends et al. [29].

**Table 2:** Percentages of amino acidic composition, IP, HBs and SBs for amylomaltases from extremophiles. Only the names of species are given, using the same background colours of Table 1. Accession numbers are reported in Table 1. The last two species correspond to amylomaltases with a *N*-terminal extrasequence. Amino acids are reported according to increasing IP values. The sum of percentages for acidic amino acids (Asp+Glu) and basic amino acids (Arg+Lys) is also given. For each amino acid, the highest (green) and lowest (red) values are reported in bold.

	*P. aerophylum/H*	*P. calidifontis/H*	*T. uzoniensis/T*	*H. walsbi/Ha*	*A. aeolics/H*	*D. geothermalis/T*	*T. aquaticus/T*	*T. brockianus/T*	*T. filiformis/T*	*T. scotoductus/T*	*T. thermophilus/T (Q5SIV3)*	*T. thermophilus/T (O87172)*	*S. agalactiae*	*A. cellulotycus/T*	*C. glutamicum*
Asp	5.1	5.1	5.8	**11.2**	4.3	6.4	4.2	4.0	4.9	**3.6**	4.0	4.0	8.0	7.3	7.4
Glu	6.6	6.8	7.3	**5.5**	**11.8**	6.4	9.0	10.4	9.3	10.2	10.0	10.0	8.0	**5.5**	7.8
Cys	0.9	0.9	**0.0**	0.9	0.2	**0.0**	0.4	0.4	0.4	0.2	0.2	0.2	0.2	0.9	**1.0**
Asn	3.6	3.4	2.9	2.9	4.3	3.0	1.6	1.8	1.6	1.6	1.6	1.6	**4.6**	**1.2**	3.4
Phe	4.1	3.4	4.6	4.2	**7.4**	6.2	5.8	6.4	5.8	6.4	6.2	6.2	5.8	**2.3**	3.0
Thr	3.8	4.3	2.5	5.7	**2.3**	4.8	2.8	3.4	2.9	3.0	3.0	3.0	**6.4**	3.4	5.0
Ser	3.8	**2.8**	4.0	**6.4**	4.1	3.2	**2.8**	3.0	2.9	3.0	**2.8**	**2.8**	4.0	3.3	5.4
Tyr	**5.6**	5.1	5.2	4.6	**5.6**	3.6	3.8	3.4	3.7	3.8	3.6	3.6	5.0	**2.2**	2.4
Met	1.5	1.9	1.7	**0.9**	1.0	2.0	1.8	1.6	1.6	2.2	2.0	2.0	**2.8**	1.0	2.1
Gln	1.9	2.6	1.9	4.2	**1.4**	**4.8**	3.0	2.2	3.3	2.8	2.2	2.0	4.0	3.0	4.1
Trp	4.3	3.8	4.0	3.1	3.1	3.8	4.4	4.4	4.3	**4.8**	4.2	4.2	2.8	2.7	**2.0**
Gly	8.1	8.8	8.5	7.0	**6.4**	8.7	8.8	9.0	8.7	**9.6**	9.0	9.0	6.8	8.0	6.8
Ala	9.4	9.6	9.8	7.2	**4.1**	10.9	11.2	10.6	11.3	9.8	10.8	10.8	6.4	**17.4**	9.8
Val	**8.3**	6.6	5.6	6.4	5.2	4.6	5.6	5.2	5.6	5.2	5.8	5.8	**4.2**	8.0	6.7
Leu	8.5	9.8	11.0	8.6	12.2	9.9	11.6	10.8	**12.4**	10.6	10.8	10.8	**7.6**	10.3	10.6
Ile	2.4	2.1	3.5	4.8	4.3	3.4	2.4	2.8	**1.9**	2.2	2.2	2.2	**6.6**	2.7	5.0
Pro	7.3	**7.9**	6.4	6.2	4.9	6.4	7.6	7.2	7.0	7.4	7.6	7.6	**4.0**	7.7	6.2
His	2.4	3.0	1.9	**3.7**	2.1	3.4	2.6	2.6	**1.6**	2.8	2.6	2.6	**1.6**	2.0	2.8
Lys	5.1	3.2	2.7	1.1	**8.0**	1.6	3.8	2.6	1.9	2.2	3.4	3.4	6.2	**0.5**	1.7
Arg	7.3	8.8	**10.8**	5.1	7.2	7.2	6.8	8.2	8.9	8.6	8.0	8.2	**4.6**	10.6	6.9
Asp+Glu	**11.8**	12.0	13.1	**16.7**	16.1	12.7	13.2	14.4	14.2	13.8	14.0	14.0	16.1	12.8	15.3
Arg+Lys	12.4	12.0	13.5	**6.3**	**15.3**	8.8	10.7	10.8	10.7	10.8	11.4	11.6	10.8	11.1	8.6
IP	8.41	7.25	8.46	4.35	6.30	5.28	5.58	5.31	5.11	5.52	5.60	5.67	4.80	5.72	4.75
HB-M	162	161	165	241	158	244	248	225	238	249	249	249	201	305	327
HB_S	5	10	9	0	12	14	29	26	21	29	30	30	20	14	57
SB	8	6	6	7	14	5	15	19	10	17	19	19	18	9	26

Legend: IP, isoelectric point; HB-M, hydrogen bonds among main chain atoms; HB-S, hydrogen bonds among side chain atoms; SB, salt bridges.

## Data Availability

Not applicable.

## Supplementary Materials

The following are available online at https://www.mdpi.com/article/10.3390/biom11091335/s1, Table S1: Percent identity matrix; Table S2: Amino acids of conserved regions in amylomaltases.
