# Supplementary material for: Amylomaltases in Extremophilic Microorganisms
Source: Biomolecules. 2021 Sep 9;11(9):1335. doi: 10.3390/biom11091335 (PMC8465469; doi:10.3390/biom11091335)
Supplement: Supplementary file 1 [file biomolecules-11-01335-s001.zip › biomolecules-1305501-supplementary.pdf]

Supplementary Material

Amylomaltases in Extremophilic Microorganisms

Claudia Leoni [1], Bruno A.R. Gattulli [1], Graziano Pesole [1,2], Luigi R. Ceci [1,*] and Mariateresa Volpicella [1,2,*]

[1] Institute of Biomembranes, Bioenergetics and Molecular Biotechnologies, Consiglio Nazionale delle Ricerche, Via Amendola, 70126 Bari, Italy; c.leoni@ibiom.cnr.it (C.L.); b.gattulli@ibiom.cnr.it (B.A.R.G.); graziano.pesole@uniba.it (G.P.)

[2] Department of Biosciences, Biotechnologies and Biopharmaceutics, University of Bari Aldo Moro, Via Amendola 165/A, 70126 Bari, Italy

* Correspondence: l.ceci@ibiom.cnr.it, Tel.: +39-080-544-3311 (L.R.C.); mariateresa.volpicella@uniba.it, Tel.: +39-080-544-3311 (M.V.)

Content

- Table S1: Percent Identity Matrix.
- Table S2: Amino acids of conserved regions in amylomaltases.

Table S1. Percent Identity Matrix

	1	2	3	4	5	6	7	8	9	10	11
1: Q1J0L5	100.00	53.65	55.87	55.26	56.48	56.48	56.07	44.61	47.28	45.16	45.16
2: A0A0K0Q020		100.00	72.78	70.52	72.58	72.58	71.55	46.30	48.62	46.84	46.41
3: Q2VJA0			100.00	83.40	86.80	86.80	86.40	46.67	47.38	47.20	45.47
4: Q6JHX9				100.00	87.40	87.40	84.60	45.83	46.96	47.84	45.26
5: Q5SIV3					100.00	99.80	88.00	46.46	47.38	47.20	45.26
6: O87172						100.00	88.00	46.46	47.38	47.20	45.26
7: A0A430UEB1							100.00	45.21	47.59	47.84	45.91
8: O66937								100.00	46.86	46.67	45.59
9: F2L2Q5									100.00	62.63	58.84
10: A3MU77										100.00	75.11
11: Q8ZXM0											100.00

Percent Identity Matrix, as obtained by Clustal 2.1. In red identity values among archaeal and bacterial sequences.

Table S2. Amino acids of conserved regions in amylomaltases. Only some representative bacterial (cyan background) and archaeal (green background) enzymes have been reported. Entries with a white background are related to enzymes from mesophilic *Bacteria* used for comparison in this review. Letters after the name of the organism refer to its classification as Hyperthermophiles (H), Thermophiles (T), Halophiles (Ha) or Mesophiles (M).

	O87172	Q8ZXM0	MZ422727	A0A0E1EIJ0	A0LVB3	Q8NNA7
Organism	*T. thermophilus*/T	*P. aerophilum*/H	*H. walsbyi*/Ha	*S. agalactiae*/M	*A. cellulolyticus*/T	*C. glutamicum*/M
REGION						
ACTIVE SITE						
i) Catalytic residues	D293	D271	D345	D295	D467	D460
	E340	E318	E392	E342	E515	E508
	D395	D371	D445	D396	D567	D561
ii) Four conserved amino acids for substrate binding	Y59	Y59	Y61	Y58	--	--
	D213	D191	D266	D215	D393	D386
	R291	R269	R343	R293	R465	R458
	H394	H370	H444	H395	H566	H560
iii) Other conserved amino acids of the active site	F217	Y195	Y270	Y219	G397	G390
	W258	W236	W310	W260	W432	W425
	H294	H272	H346	H296	H468	H461
	L342	L320	L394	L344	L517	L510
	N464	N434	N508	N460	N715	N661
iv) Conserved amino acids forming part of the cleft around the active center	S57	S57	S59	S56	--	--
	P58	P58	P60	P57	--	--
	D341	D319	D393	N343	D516	D516
	G343	G321	G395	G345	G518	G518
	T393	T369	T443	T394	T565	T565
	P466	P436	P510	P462	P717	P717
250s LOOP	P247	P225	P300	P249	P421	P414
	P248	P226	P301	A250	A422	P415
	D249	D227	T302	D251	D423	D416
	Y250	Y228	D303	D252	M424	G417
	F251	F229	--	F253	Y425	Y418
	S252	S230	S304	S254	N426	N419
	E253	A231	D305	D255	Q427	Q420
	T254	T232	D306	D256	Q428	Q421
	G255	G233	G307	G257	G429	G422
	W302	W280	Y354	W304	W476	F469
SECONDARY SUBSTRATE BINDING SITE	Y54	Y54	H56	F53	--	--
	Y101	W101	R107	F105	W280	Y290
	Y465	R435	E509	M461	Q716	Q662
